# Allostatic load as a predictor of postoperative complications in patients with breast cancer

**DOI:** 10.1038/s41523-024-00654-2

**Published:** 2024-06-12

**Authors:** J. C. Chen, Mohamed I. Elsaid, Demond Handley, Lisa Anderson, Barbara L. Andersen, William E. Carson, Joal D. Beane, Alex Kim, Roman Skoracki, Timothy M. Pawlik, Samilia Obeng-Gyasi

**Affiliations:** 1 Division of Surgical Oncology, Department of Surgery, The Ohio State University, Columbus, OH USA; 2 Department of Biomedical Informatics, College of Medicine, The Ohio State University, Columbus, OH USA; 3 Center for Biostatistics, College of Medicine, The Ohio State University, Columbus, OH USA; 4 Division of Medical Oncology, Department of Internal Medicine, The Ohio State University, Columbus, OH USA; 5 Department of Psychology, The Ohio State University, Columbus, OH USA; 6 Division of Reconstructive Oncologic Plastic Surgery, Department of Plastic Surgery, The Ohio State University, Columbus, OH USA

**Keywords:** Breast cancer, Predictive markers, Outcomes research

## Abstract

Allostatic load (AL) is a biological measure of cumulative exposure to socioenvironmental stressors (e.g., poverty). This study aims to examine the association between allostatic load (AL) and postoperative complications (POC) among patients with breast cancer. Females ages 18+ with stage I-III breast cancer who received surgical management between 01/01/2012-12/31/2020 were identified in the Ohio State Cancer registry. The composite AL measure included biomarkers from the cardiovascular, metabolic, immune, and renal systems. High AL was defined as composite scores greater than the cohort’s median (2.0). POC within 30 days of surgery were examined. Univariable and multivariable regression analysis examined the association between AL and POC. Among 4459 patients, 8.2% had POC. A higher percentage of patients with POC were unpartnered (POC 44.7% vs no POC 35.5%), government-insured (POC 48.2% vs no POC 38.3%) and had multiple comorbidities (POC 32% vs no POC 20%). Patients who developed POC were more likely to have undergone sentinel lymph node biopsy followed by axillary lymph node dissection (POC 51.2% vs no POC 44.6%). High AL was associated with 29% higher odds of POC (aOR 1.29, 95% CI 1.01–1.63). A one-point increase in AL was associated with 8% higher odds of POC (aOR 1.08, 95% CI 1.02-1.16) and a quartile increase in AL was associated with 13% increased odds of POC (aOR 1.13, 95% CI 1.01–1.26). Among patients undergoing breast cancer surgery, increased exposure to adverse socioenvironmental stressors, operationalized as AL, was associated with higher odds of postoperative complications.

## Introduction

The recognition that breast cancer is a local and systemic disease has resulted in significant changes in the surgical management of breast cancer^1^. Specifically, the approach to surgical treatment for early-stage breast cancer has shifted from performing extensive procedures like radical mastectomy towards adopting more minimally invasive techniques, such as breast-conserving surgery^1^. Further, in clinically node-negative patients, axillary management has deescalated from routine axillary lymph node dissection (ALND) to sentinel lymph node biopsy (SLNB) or omission of lymph node surgery in some populations^2,3^. Consequently, morbidity and mortality among patients undergoing breast and axillary surgery have improved with a lower incidence of complications^4,5^.

Nevertheless, patients from marginalized and minoritized groups continue to experience high postoperative complication (POC) rates and lower quality-of-life compared to individuals from well-resourced groups. For example, Black women undergoing breast surgery are more likely to have longer lengths of stay, develop POCs, and experience higher in-hospital mortality than White women^6,7^. Similarly, patients living in areas of high deprivation report poorer psychosocial well-being and physical functioning after breast surgery than their counterparts living in areas with less deprivation^8^. A plausible explanation for these racial and socioeconomic disparities in postoperative outcomes is an interplay between greater rates of comorbidities and higher socioenvironmental stressors (e.g., low socioeconomic status) often experienced by marginalized and minoritized women^9,10^.

**Fig. 1:**
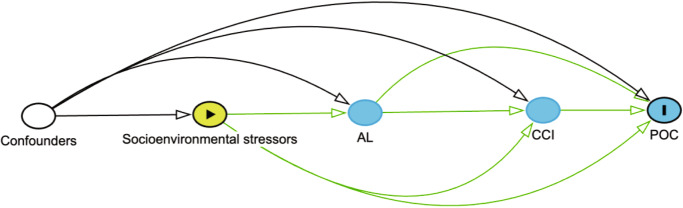
Examination of the relationship between allostatic load and postoperative complications. Hypothesized causal diagram illustrating the mediation effects of allostatic load (AL) and the Charlson Comorbidity Index (CCI) on the relationship between socioenvironmental stressors and the development of postoperative complications (POC).

In this study, we examine the relationship between biological correlates of exposure to socioenvironmental stressors, operationalized as allostatic load, on POC among patients with breast cancer who receive surgical treatment. Allostatic load (AL) is a measure of physiologic dysregulation secondary to exposure to stressful socioenvironmental stimuli (e.g., low socioeconomic status)^11^. AL is derived from a combination of primary mediators (e.g., cortisol), secondary outcomes (e.g., glucose) and tertiary outcomes (e.g., diabetes). Our prior work demonstrated that patients with breast cancer who were racialized as Black, unpartnered, insured with Medicaid, and had higher Charlson Comorbidity Indices (CCI) were more likely to have high AL than White, privately insured individuals without comorbidities^10^. Similarly, patients with lung cancer who had lower educational achievement, limited mobility, poor self-care, depressive symptoms, and multiple stressful life events had higher AL^12^. Moreover, patients with breast or lung cancer with high AL had worse all-cause mortality relative to patients with low AL^10,12^. Collectively, these studies suggest AL may serve as a pathway to elucidate the relationship between socioenvironmental stressors and POC (Fig. 1) beyond consideration of only medical comorbidities. The objective of the current study was to examine the association between AL and POC. We hypothesized that patients with high AL at diagnosis would have a higher probability of experiencing POC.

## Results

### Patient characteristics

Among 4459 patients in the analytic cohort, 365 (8.2%) developed POC (Table 1). Patients who developed POC were more likely to be unpartnered (single 17.3% vs 14.1%, widowed/separated/divorced 27.4% vs 21.4%, *p* = 0.002) and have government insurance (Medicaid 35.6% vs 30.2%, Medicare 12.6% vs 8.1%, *p* < 0.001). A higher proportion of patients who experienced POC had ≥1 comorbidity (32.1% vs 20.1%, *p* < 0.001). Patients who developed POC were more likely to have undergone SLNB followed by ALND (51.2% vs 44.6%, *p* = 0.015) but were less likely to have had reconstructive surgery (21.4% vs 26.3%, *p* = 0.038). There were no differences in the type of breast surgery (lumpectomy vs mastectomy) or receipt of chemotherapy, hormone therapy, or radiation therapy (*p* > 0.05). Most notably, patients who developed POC had a higher AL at diagnosis (58.4% vs 48.6%, *p* < 0.001) than those with no POC. Patient characteristics stratified by AL status are summarized in Supplementary Table 1.

**Table 1:** Overview of sociodemographic and clinical characteristics by postoperative surgical complications status^a^

Patient characteristic	All	Postoperative Surgical Complications		*P*-Value^b^
		Yes	No	
	*n* = (4459)	*n* = (365)	*n* = (4094)	
**Age group,** ***n*** **(%)**				0.103
≤39	313 (7)	17 (4.7)	296 (7.2)	
40–49	838 (18.8)	70 (19.2)	768 (18.8)	
50–59	1184 (26.6)	85 (23.3)	1099 (26.8)	
60–59	1286 (28.8)	112 (30.7)	1174 (28.7)	
70+	838 (18.8)	81 (22.2)	757 (18.5)	
**Race-ethnicity**, ***n*** **(%)**				0.095
Hispanic-Black	3 (0.1)	0 (0)	3 (0.1)	
Non-hispanic-Black	381 (8.5)	46 (12.6)	335 (8.2)	
Hispanic-White	23 (0.5)	1 (0.3)	22 (0.5)	
Non-hispanic-White	3861 (86.6)	302 (82.7)	3559 (86.9)	
Hispanic-other	27 (0.6)	3 (0.8)	24 (0.6)	
Non-hispanic-other	164 (3.7)	13 (3.6)	151 (3.7)	
**Marital status,** ***n*** **(%)**				0.002
Single	639 (14.3)	63 (17.3)	576 (14.1)	
Married/living as married	2843 (63.8)	202 (55.3)	2641 (64.5)	
Widowed, separated or divorced	977 (21.9)	100 (27.4)	877 (21.4)	
**Health insurance,** ***n*** **(%)**				<0.001
Managed care	2650 (59.4)	184 (50.4)	2466 (60.2)	
Medicaid	1365 (30.6)	130 (35.6)	1235 (30.2)	
Medicare	376 (8.4)	46 (12.6)	330 (8.1)	
Other	68 (1.5)	5 (1.4)	63 (1.5)	
**Smoking history,** ***n*** **(%)**				0.082
Never	2787 (62.5)	211 (57.8)	2576 (62.9)	
Current or former	1672 (37.5)	154 (42.2)	1518 (37.1)	
**Alcohol use,** ***n*** **(%)**				0.029
Never	2103 (47.2)	192 (52.6)	1911 (46.7)	
Current or former	2356 (52.8)	173 (47.4)	2183 (53.3)	
**Charlson comorbidity index**^**c**^, ***n*** **(%)**				<0.001
0	3520 (78.9)	248 (67.9)	3272 (79.9)	
1–3	826 (18.5)	92 (25.2)	734 (17.9)	
4+	113 (2.5)	25 (6.8)	88 (2.1)	
**HER-2+ summary,** ***n*** **(%)**	692 (15.5)	59 (16.2)	633 (15.5)	0.703
**Progesterone+ summary,** ***n*** **(%)**	3111 (69.8)	255 (69.9)	2856 (69.8)	0.967
**Estrogen+ summary,** ***n*** **(%)**	3569 (80.0)	288 (78.9)	3281 (80.1)	0.571
**Molecular subtype,** ***n*** **(%)**				0.796
Hormone receptor negative/HER 2 positive	242 (5.4)	24 (6.6)	218 (5.3)	
Hormone receptor positive/HER 2 negative	2753 (61.7)	223 (61.1)	2530 (61.8)	
Hormone receptor positive/HER2 positive	818 (18.3)	66 (18.1)	752 (18.4)	
Hormone receptor negative/HER negative	646 (14.5)	52 (14.2)	594 (14.5)	
**Cancer stage,** ***n*** **(%)**				0.206
1	2814 (63.1)	211 (57.8)	2603 (63.6)	
2	1369 (30.7)	121 (33.2)	1248 (30.5)	
3	276 (6.2)	33 (9.0)	243 (5.9)	
**Mastectomy,** ***n*** **(%)**	2124 (47.6)	187 (51.2)	1937 (47.3)	0.151
**Lumpectomy,** ***n*** **(%)**	2306 (51.7)	201 (55.1)	2105 (51.4)	0.180
**Sentinel lymph node biopsy only,** ***n*** **(%)**	1444 (32.4)	94 (25.8)	1350 (33.0)	0.005
**Axillary lymph node biopsy only,** ***n*** **(%)**	237 (5.3)	20 (5.5)	217 (5.3)	0.884
**Both sentinel and axillary lymph node biopsies,** ***n*** **(%)**	2013 (45.1)	187 (51.2)	1826 (44.6)	0.015
**Reconstructive surgery**	1156 (25.9)	78 (21.4)	1078 (26.3)	0.038
**Hormone therapy,** ***n*** **(%)**	3355 (75.2)	272 (74.5)	3083 (75.3)	0.739
**Radiation therapy,** ***n*** **(%)**	2679 (60.1)	216 (59.2)	2463 (60.2)	0.713
**Chemotherapy,** ***n*** **(%)**	2112 (47.4)	184 (50.4)	1928 (47.1)	0.224
**High allostatic load,** ***n*** **(%)**	2202 (49.4)	213 (58.4)	1989 (48.6)	<0.001

^a^Either technical, cardiovascular, respiratory, urinary, or infectious postoperative complications

^b^*P*-value from Wilcoxon rank sum test for age but *p*-values from Chi-Square tests to test the association between postoperative complications and other patient characteristics

^c^Using Charlson Comorbidity Index weights (excluding cancer)

### Relationship between AL and postoperative complications

**Fig. 2:**
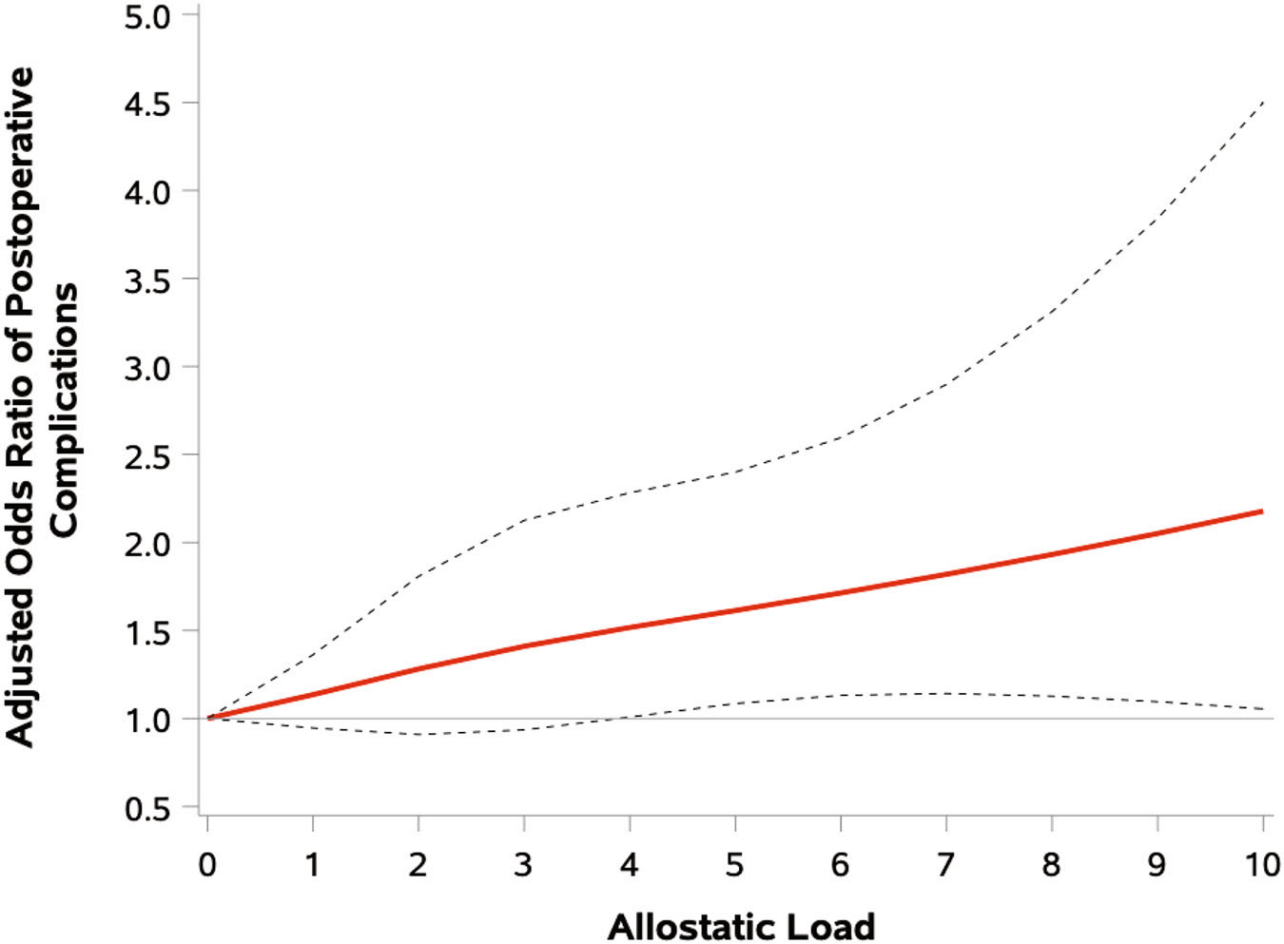
Examination of the relationship between allostatic load and postoperative complications. Adjusted Odds Ratios (OR) for the relationship between allostatic load and development of postoperative complications.

Patients with high AL had 48% higher odds of developing POC (OR 1.48, 95% CI: 1.18–1.86), which remained significant after adjusting for sociodemographic, clinical, and treatment factors (aOR 1.29, 95% CI: 1.01–1.63) (Table 2). The odds of developing a POC increased by 8% for every one unit increase in AL (aOR 1.08, 95% CI: 1.02 to 1.16); there was 13% increased odds of developing a POC for every one quartile increase in AL (aOR 1.13, 95% CI: 1.01–1.26). There was a linear dose-response relationship in the association between increasing AL and POC development (Fig. 2), which was significant when the adjusted composite AL was ≥ 5 (Supplementary Table 2). On sub-analyses, albumin was the primary biomarker associated with development of POC in both univariate and adjusted analysis (aOR 2.73, 95% CI: 1.34–5.52) **(**Supplementary Table 3).

**Table 2:** Crude and Adjusted^a^ Association between High Allostatic Load and Postoperative Complications

Exposure	Crude	χ2 statistic, DF, *p*-value	Adjusted	χ2 statistic, DF, *p*-value
	OR (95%CI)		OR (95%CI)	
**Allostatic load**^**b**^				
High	1.48 (1.18–1.86)	11.6, 1, *p* < 0.001	1.29 (1.01–1.63)	103.7, 29, *p* < 0.001
Low	Ref.		Ref.	
**Per unit increase in allostatic load**	1.13 (1.07–1.21)	15.3, 1, *p* < 0.001	1.08 (1.02–1.16)	105.2, 29, *p* < 0.001
**Per quartile increase in allostatic load**^**c**^	1.21 (1.09–1.35)	11.7, 1, *p* < 0.001	1.13 (1.01–1.26)	103.4, 29, *p* < 0.001

^a^Logistic regression model adjusted for age, race, ethnicity, health insurance, marital status, history of alcohol use and smoking, molecular subtype, AJCC clinical stage, lumpectomy, mastectomy, reconstructive surgery, sentinel and axillary lymph node biopsy, and receipt of chemotherapy.

^b^High allostatic load > median sum allostatic load score.

^c^Allostatic load per one increase in sum allostatic load score.

*OR* Odds Ratio, *CI* Confidence Interval.

In the exploratory mediation analysis, the adjusted total effect of AL on POC was OR 1.15 (95% CI: 1.03–1.28) per quartile increase in AL^13^. An estimated 32.1% (95% CI: 4.4–59.6%) of the adjusted effect of AL on POC was potentially mediated through the development of chronic comorbidities, while 69.9% (95% CI: 40.2–95.6%) of the adjusted effect of AL on POC was potentially due to the direct association between AL and POC.

## Discussion

While previous evidence has suggested a relationship between socioenvironmental stressors and postoperative outcomes, the current study is the first to evaluate the relationship between biological correlates of internalized stress, operationalized as AL, and the development of POC. Amongst the patients included in this study, high AL at time of diagnosis was associated with a higher probability of developing POC. Specifically, there was a linear relationship between increasing AL and the development of POC. Moreover, exploratory analysis suggests that AL may impact the association between socioenvironmental stressors and POC both directly and indirectly through comorbidities.

An important finding of the current study was that AL may be predictive of POC. Compared to comorbidity-based indices, the use of peripheral biomarkers relies on more objective data rather than self-reported chronic medical conditions. Further, a diagnosis of a medical comorbidity requires sufficient accrual of physiologic dysregulation to produce the clinical manifestation of disease, serving as the “end product” of malfunctional adaptation^14^. AL, however, measures the primary chemical messengers that produce the downstream physiologic dysregulation ultimately leading to disease manifestation^15^. AL may thereby be more sensitive to detect subclinical processes preceding the development of comorbidities^14^. Additionally, AL may incorporate the influence of protective factors and unhealthy coping behaviors used to compensate for the physiologic dysregulation, which is excluded when considering comorbidities alone^14^.

AL biomarkers are hypothesized to follow a bifactor model, suggesting that the combination of biomarkers represents both a common factor (i.e., allostatic load) underlying system-wide physiologic dysfunction, but also unique, system-specific effects^16,17^. Essentially, AL examines both shared and system-specific effects, allowing for greater precision to evaluate the effects of socioenvironmental stressors on physiologic dysfunction. Furthermore, AL biomarkers exhibit parameter invariance, suggesting the comparability of derived AL scores even when the exact subset of biomarkers varies^16^. Comorbidity-based indices such as CCI use a weighted index to take into account the number and severity of comorbidities based on the adjusted hazard risk of 2 year noncancer inpatient mortality^18,19^. Conditions that may significantly influence mortality in the outpatient setting are excluded and the discriminatory ability of comorbidities for outcome predictions decreases with age^19,20^. Additionally, disease severity and degree of disease control with treatment are ignored. In contrast, some studies suggest AL remains a significant predictor of all-cause and cancer-specific mortality amongst older patients^21^.

Similar to prior studies, patients with more comorbidities in the current cohort were more likely to develop POC^22^. A plethora of evidence has noted associations between the Charlson Comorbidity Index, currently considered a gold-standard measure to assess the influence of comorbidities in clinical research, and the development of POC in a myriad of conditions, including breast cancer^18,23–26^. Prior systematic reviews have evaluated the influence of individual factors such as age, sex assigned at birth, and socioeconomic status on multimorbidity, defined as the presence of more than one health condition^27^. Most recently, Alvarez-Galvez et al categorized the impact of six domains on the risk of multimorbidity: individual sociodemographic factors, socioeconomic status, lifestyle behaviors, social networks and social relationships, residential characteristics, and health service usage^28^. Specifically, Alvarez-Galvez et al noted that individuals with lower educational levels, lower income, racialized as Black, Native American, or Asian, who resided in areas with higher economic deprivation and poorer social networks had a greater risk of suffering from multimorbidity^28^. This chronic socioenvironmental adversity is similarly suggested to lead to the persistent activation of the hypothalamic-pituitary-adrenal (HPA) axis and sympathetic adrenal medullary (SAM) pathway that underscores the theoretical framework for allostatic load^29–31^. As such, AL may serve as a plausible mechanistic pathway between socioenvironmental stressors and the development of POC. For instance, exploratory mediation analysis in the current study suggested AL at diagnosis may predict POC while concomitantly sharing a potential causal pathway with multimorbidity, indicating the possibility that AL may capture mechanisms impacting patients’ clinical courses in ways that are not fully accounted for when solely considering comorbidities. Nevertheless, the mediation analysis results should be interpreted with caution as the cross-sectional nature of the data limits causal interpretations.

Although CCI is currently one of the most widely used assessments when considering surgical morbidity and mortality, it may not be the optimal approach to measure the impact of socioenvironmental stressors on the development of POC. CCI often relies on International Classification of Disease (ICD) codes, which not only require adequately integrated healthcare systems but necessitate accurate ICD coding^19^. A comprehensive review evaluating ICD-9 code accuracy in representing the clinical presence or absence of a chronic condition noted that 80% of conditions had positive predictive values and negative predictive values of at least 70%, but with marked variation ranging from 9 to 100%^32^. Reliance on self-reported chronic medical conditions would similarly underestimate the prevalence of chronic illnesses^33^. Moreover, use of the CCI relies on adequate healthcare utilization to ensure appropriate screening. However, low healthcare utilization is pervasive, particularly among current and historically marginalized communities (i.e., racialized minorities, especially the Black community) with greater mistrust of the healthcare system^33,34^. These limitations may lead to differential misclassification of patients who may otherwise benefit from preventative services, further widening the health disparities gap.

In the current study, patients who developed POC were also more likely to be unpartnered and government insured. Few studies have previously examined the relationship between marital status and the development of POC amongst patients with breast cancer. However, existing studies suggest a decreased risk of cancer-specific and all-cause mortality amongst married women with breast cancer relative to their unpartnered counterparts^35,36^. The impact of marital status on POC development varies among other cancer types; divorced or separated patients with oropharyngeal or laryngeal cancer have twice the odds of requiring readmission for complications but no association is seen amongst patients with colorectal cancer^37,38^. Yu et al noted that patients with breast cancer with government insurance, particularly Medicare, were also more likely to develop POC even after controlling for age and comorbidities^39^. Additional work is needed to determine the pathways between insurance, marital status, and POC. Of note, there were no racial differences among patients who did versus did not experience POC.

The biomarkers used in our composite AL score were routinely collected during the pre-operative clinic visit and prior to any surgical intervention^40^. As such, incorporation of AL for risk stratification in clinical practice is feasible. Additionally, using biomarkers commonly collected as part of the pre-operative breast cancer workup standardizes care across all individuals, which may provide opportunities to improve disparities in cancer care^41,42^.

Our exploratory analysis suggests that AL and comorbidities may share a causal pathway to the development of POC. However, lack of temporality limits interpretation of these findings. The low incidence of POC development amongst patients with breast cancer decreases our ability to detect differences in sociodemographic and clinical features, potentially creating bias towards the null. Additionally, the results of this single institution study may not be generalizable to other practices. Regardless, our findings suggest an alternative method of evaluating the risk of developing POC while simultaneously providing an avenue to standardize care and provide further opportunities to decrease the disparity gap.

Indices incorporating comorbidities have become the gold standard method to evaluate the influence of comorbidities on clinical outcomes, including postoperative complications. However, use of comorbidities requires well-integrated healthcare systems, accurate coding, and adequate healthcare utilization. The current study demonstrated that allostatic load, an objective measure of cumulative stress from socioenvironmental factors, may predict the development of postoperative complications. Assessment of allostatic load may thereby serve as an opportunity to standardize care and provide opportunities to decrease disparities.

## Methods

### Data source

Self-reported females ≥18 years old initially diagnosed with stage I-III breast cancer between 01/01/2012-12/31/2020 who received surgical management at the Ohio State University James Comprehensive Cancer Center were identified through the Cancer Center’s Registry (Supplementary Fig. 1). Patients with ductal carcinoma in-situ (stage 0), metastatic disease (stage IV), recurrent breast cancer, unknown breast cancer subtype, or those who did not receive surgical treatment were excluded. Surgical treatment was considered an inclusion criteria as (1) most patients with stage I-III breast cancer undergo surgical treatment and (2) biomarkers used to calculate AL are part of the pre-operative workup^43^.

### Sociodemographic variables

Sociodemographic variables studied were age, race (White, Black, Other), ethnicity (Hispanic or non-Hispanic), marital status (single, married/living as married, widowed/separated/divorced), health insurance (managed care, Medicaid, Medicare, other), and smoking and alcohol histories (never, current/former). Patients who identified as Asian, American Indian, Alaskan Native, Native Hawaiians, other Pacific Islander, or multiracial were categorized into the “Other” racial category due to small sample sizes. Racial categories in this study are a social construct and not a reflection of genetic ancestry^44^.

### Clinical and treatment characteristics

Patient hormone receptor status [estrogen (ER), progesterone (PR), ERBB2 expression (HER2)], and cancer stage were obtained. Patients were then categorized into molecular subgroups: hormone receptor (HR) negative/ERBB2 positive, HR + /ERBB2-, HR + /ERBB2 + , or HR-/ERBB2-. Cancer treatment included breast surgery (lumpectomy vs mastectomy) and axillary (sentinel lymph node biopsy (SLNB) vs axillary lymph node dissection (ALND)) surgery, breast reconstruction (yes/no), receipt of systemic therapy (hormone therapy (yes/no), chemotherapy (yes/no)), and radiation therapy (yes/no).

### Study measures

#### Allostatic load (AL)

Although there is no universally accepted standard for AL biomarkers, multisystem modeling has determined that factor loadings remain consistent as long as biomarkers from various physiological systems are incorporated^11,16^. The composite AL measure was created using biomarkers routinely collected as part of the pre-operative workup for breast cancer surgery. Specifically, biomarkers from the cardiovascular (i.e., heart rate (HR), systolic (SBP) and diastolic (DBP) blood pressure), metabolic (i.e., body mass index (BMI), alkaline phosphatase (ALP), blood glucose, albumin), immune (i.e., white blood cell count; WBC), and renal (i.e., blood urea nitrogen, BUN; creatinine) systems were used. Biomarkers collected up to 12 months before or 6 months after biopsy-proven breast cancer diagnosis were retrieved from electronic medical records. Biomarker distributions were evaluated within the cohort. Each biomarker in the worst quartile was assigned one point. For example, values ≥75th percentile for HR, SBP, DBP, BMI, ALP, glucose, WBC, creatinine, and BUN were each given a point. Similarly, values ≤25th percentile for albumin were assigned a point. For each individual, points were summed for a composite AL score ranging from 0–10. Composite scores were then dichotomized into high versus low AL using the cohort’s median score (2.0) as the cutoff. Higher AL is indicative of worse physiologic dysregulation.

### Study outcome

The primary study outcome was the development of a post-operative complication (POC) within 30 days of surgery, which are listed in Supplementary Table 4. Development of a post-operative complication was dichotomized into yes or no, then categorized into technical, infectious, respiratory, cardiovascular, or urinary complications.

### Statistical analysis

All missing values were imputed using multiple imputations by chained equations to create ten imputed data sets^45^. Auxiliary and participant characteristics associated with the missing patterns of each imputed variable were included and all imputation-corrected parameters and standard errors were combined using Rubin’s method^46^.

Sociodemographic characteristics were summarized using descriptive statistics, including means and standard deviations (SD) for continuous variables and frequencies and proportions for categorical variables. Differences between patients with and without POC were compared using the Wilcoxon rank-sum test for continuous variables and χ^2^ or Fisher’s exact tests for categorical variables.

Crude and adjusted logistic regression models with robust standard errors were used to assess the association between POC as the outcome and AL status as exposure. Additionally, dose-response relationships between the cumulative AL score in its continuous form and the odds of POC were evaluated using a three-knot restricted cubic spline in the adjusted logistic regression models. The three knots were placed at the AL sum scores of the 10th, 50th, and 90th percentiles^47^. Wald-Chi Square tests assessed the overall and nonlinear associations between the AL score percentiles and the odds of POC. All assumptions required for logistic regression (e.g., linearity of continuous predictors, independence of outcomes, logit as the correct link function) were satisfied.

Given the findings between POC and AL status, a secondary analysis examined the relationship between POC and each AL biomarker using established clinical cut-off values^12^. Univariate logistic regression models were fitted with each AL biomarker as the exposure to determine its effects on the odds of POC. Furthermore, an adjusted logistic regression model that included all AL biomarkers and high AL status was used to examine the utility of AL as an independent predictor of POC among patients undergoing surgery for breast cancer.

Although data on AL and chronic comorbidities were cross-sectional, an exploratory mediation analysis was conducted to assess the role of chronic comorbidities as a potential mediator in the relationship between AL and POC^13^. Chronic comorbidities was a binary variable representing patients with and without ≥1 chronic comorbidity. Adjusted logistic regression was fitted using (1) chronic comorbidities as the outcome and AL quartiles as exposure, and (2) POC as the outcome and AL, chronic comorbidities, and their interactions as exposures. Models in the causal mediation analysis were adjusted for age, molecular subtype, clinical stage, breast and axillary surgery type, receipt of reconstructive surgery, and chemotherapy. Results of the exploratory analysis should be interpreted as hypothesis generating given the cross-sectional nature of the data used. Two-sided *p*-values <0.5 were considered statistically significant. All analyses were performed using SAS software (version 9.4; SAS Institute, Cary, NC, USA). This study complied with all relevant ethical regulations and the Ohio State University Office of Responsible Research Practices’ institutional review board approved this study’s protocol (2021C0114). Informed consent was waived given the retrospective nature of this study. This study complied with all relevant ethical regulations including the Declaration of Helsinki. During the preparation of this manuscript the author(s) used ChatGPT to improve language and readability.

### Supplementary information

Supplementary tables and Figures

## Data Availability

The data used and analyzed during the current study are available from the Ohio State University Comprehensive Cancer Center on reasonable request.

## Code availability

The underlying code for this study may be made available to qualified researchers on reasonable request from the Ohio State University Comprehensive Cancer Center.
