# Supplementary material for: Allostatic load as a predictor of postoperative complications in patients with breast cancer
Source: NPJ Breast Cancer. 2024 Jun 12;10:44. doi: 10.1038/s41523-024-00654-2 (PMC11169387; doi:10.1038/s41523-024-00654-2)
Supplement: Supplementary file 1 — Supplementary tables and Figures [file 41523_2024_654_MOESM1_ESM.pdf]

Supplementary Tables and Figures

Supplementary Figure 1: Schema Outlining the Generation of the Study Sample

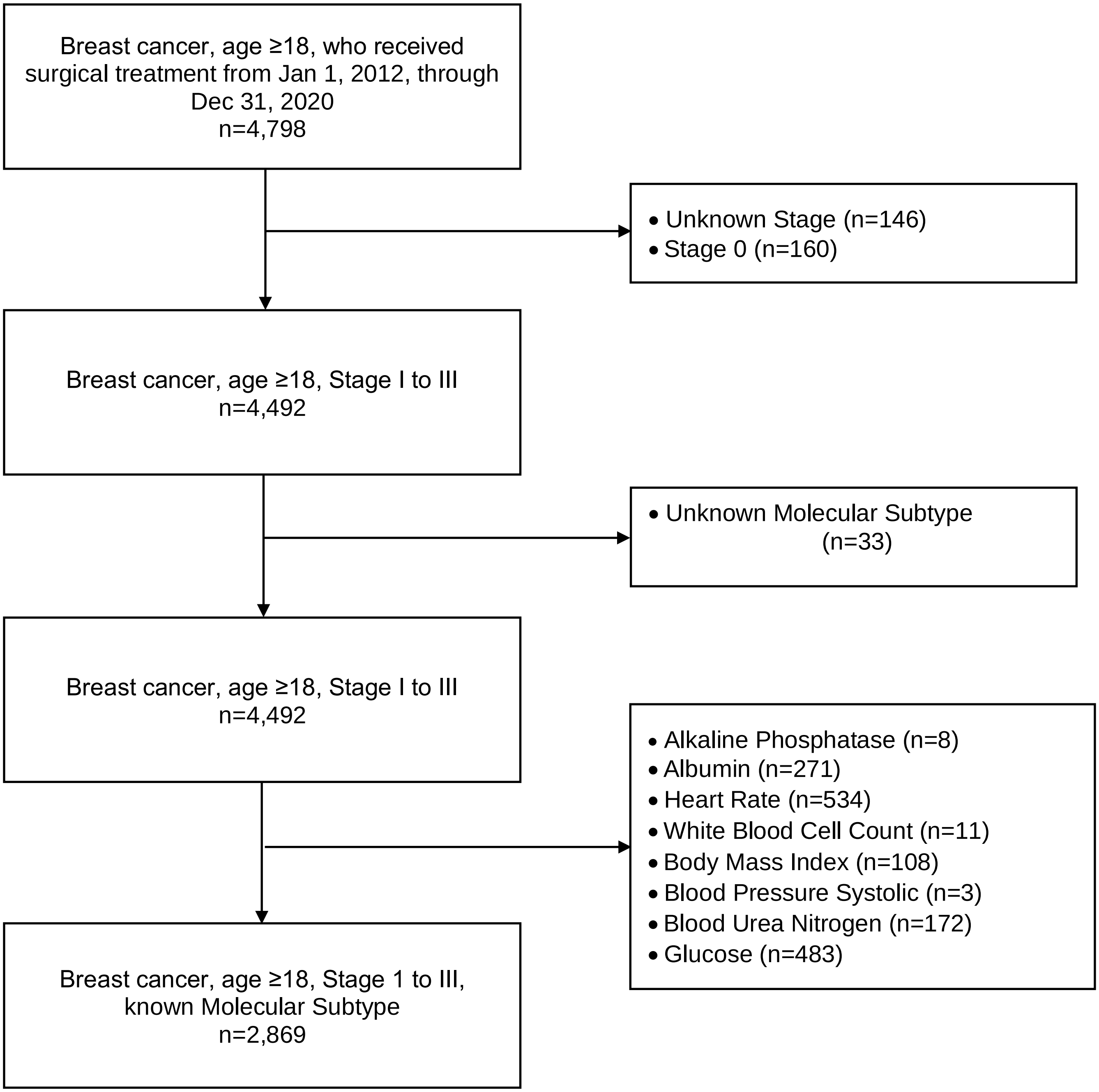

**Supplementary Table 1**. Sociodemographic and Clinical Characteristics of Patients by Allostatic Load Classification				
**Patient Characteristic**	**All**	**Low Allostatic Load**	**High Allostatic Load**	**P-Value**[a]
	**n =(4,459)**	**n =(2,257)**	**n =(2,202)**	
**Age Group, n (%)**				
≤ 39	313 (7.0)	195 (8.6)	118 (5.4)	<0.001
40 to 49	838 (18.8)	496 (22.0)	343 (15.6)	
50 to 59	1184 (26.6)	624 (27.6)	560 (25.4)	
60 to 69	1286 (28.8)	609 (27.0)	677 (30.7)	
70+	838 (18.8)	333 (14.8)	505 (22.9)	
**Race-Ethnicity, n (%)**				<0.001
Hispanic-Black	3 (0.1)	3 (0.1)	0 (0.0)	
Non-Hispanic-Black	381 (8.5)	149 (6.6)	232 (10.5)	
Hispanic-White	23 (0.5)	8 (0.4)	15 (0.7)	
Non-Hispanic-White	3861 (86.6)	1972 (87.4)	1889 (85.8)	
Hispanic-Other	27 (0.6)	19 (0.8)	8 (0.4)	
Non-Hispanic-Other	164 (3.7)	106 (4.7)	58 (2.6)	
**Marital Status, n (%)**				<0.001
Single	639 (14.3)	309 (13.7)	330 (15.0)	
Married/living as married	2843 (63.8)	1539 (68.2)	1304 (59.2)	
Widowed, Separated or Divorced	977 (21.9)	409 (18.1)	568 (25.8)	
**Health Insurance, n (%)**				<0.001
Managed Care	2650 (59.4)	1497 (66.3)	1153 (52.4)	
Medicaid	1365 (30.6)	552 (24.5)	813 (36.9)	
Medicare	376 (8.4)	172 (7.6)	204 (9.3)	
Other	68 (1.5)	37 (1.6)	32 (1.5)	
**Smoking History, n (%)**				0.124
Never	2787 (62.5)	1442 (63.9)	1345 (61.1)	
Current or Former	1672 (37.5)	815 (36.1)	858 (39.0)	
**Alcohol Use, n (%)**				<0.001
Never	2103 (47.2)	929 (41.2)	1174 (53.3)	
Current or Former	2356 (52.8)	1328 (58.8)	1028 (46.7)	
**Charlson Comorbidity Index**[b]**, n (%)**				<0.001
0	3551 (79.1)	1954 (85.9)	1597 (72.1)	<0.001
1 to 3	828 (18.4)	297 (13.1)	531 (24.0)	
4 +	113 (2.5)	25 (1.1)	89 (9.44)	
**HER-2 Summary, n (%)**				0.002
Negative	3767 (84.5)	1945 (86.2)	1822 (82.7)	
Positive	692 (15.5)	312 (13.8)	380 (17.3)	
**Progesterone Summary, n (%)**				0.395

Negative	1348 (30.2)	668 (29.6)	680 (30.9)	
Positive	3111 (69.8)	1589 (70.4)	1522 (69.1)	
**Estrogen Summary, n (%)**				0.051
Negative	890 (20.0)	423 (18.7)	467 (21.2)	
Positive	3569 (80.0)	1834 (81.3)	1735 (78.8)	
**Molecular Subtype, n (%)**				0.007
ER-/PR-/HER2+	242 (5.4)	120 (5.3)	122 (5.5)	
ER+/PR+/HER2-	2753 (61.7)	1438 (63.7)	1315 (59.7)	
ER+/PR-/HER2+	818 (18.3)	398 (17.6)	420 (19.1)	
ER-/PR-/HER2-	646 (14.5)	301 (13.3)	345 (15.7)	
**Cancer Stage, n (%)**				<0.001
1	2814 (63.1)	1494 (66.2)	1320 (59.9)	
2	1369 (30.7)	644 (28.5)	725 (32.9)	
3	276 (6.2)	119 (5.3)	157 (7.1)	
**Mastectomy, n (%)**	2124 (47.6)	1109 (49.1)	1015 (46.1)	0.055
**Lumpectomy, n (%)**	2306 (51.7)	1134 (50.2)	1172 (53.2)	0.054
**Sentinel lymph node biopsy only, n (%)**	1444 (32.4)	751 (33.3)	693 (31.5)	0.215
**Axillary lymph node biopsy only, n (%)**	237 (5.3)	110 (4.9)	127 (5.8)	0.226
**Both sentinel and axillary lymph node biopsies, n (%)**	2013 (45.1)	1046 (46.3)	967 (43.9)	0.126
**Reconstructive Surgery**	1156 (25.9)	692 (30.7)	464 (21.2)	<0.001
**Hormone Therapy, n (%)**	3355 (75.2)	1736 (76.9)	1619 (73.5)	0.013
**Radiation Therapy, n (%)**	2679 (60.1)	1331 (59.0)	1348 (61.2)	0.150
**Chemotherapy, n (%)**	2112 (47.4)	1031 (45.7)	1081 (49.1)	0.028
**Postoperative Surgical Complications, n (%)**	365 (8.2)	152 (6.7)	213 (9.7)	0.001
Technical, n (%)	233 (5.2)	111 (4.9)	122 (5.5)	<0.001
Cardiovascular, n (%)	24 (0.54)	7 (0.31)	17 (0.77)	
Respiratory, n (%)	5 (0.11)	3 (0.13)	2 (0.09)	
Urinary, n (%)	16 (0.36)	1 (0.04)	15 (0.68)	
Infectious, n (%)	87 (2.0)	30 (1.3)	57 (2.6)	

[a] P-value from Wilcoxon rank sum test for age but p-values from Chi-Square tests to test the association between postoperative complications and other patient characteristics
[b] Using Charlson Comorbidity Index weights (excluding cancer)

Supplementary Table 2. Crude and Adjusted[a] Odds Ratios of Surgical Complications Unit[b] Increase in Allostatic Load Scores Relative to Zero Allostatic Load Score (n=4,459)

Allostatic Load	Crude OR (95% CI)	Adjusted OR (95% CI)
0	Ref.	Ref.
1	1.19 (1.00 to 1.43)	1.13 (0.94 to 1.36)
2	1.41 (1.01 to 1.98)	1.27 (0.90 to 1.79)
3	1.62 (1.09 to 2.42)	1.39 (0.92 to 2.10)
4	1.81 (1.22 to 2.69)	1.49 (0.99 to 2.24)
5	2.00 (1.37 to 2.91)	1.58 (1.06 to 2.35)
6	2.20 (1.49 to 3.25)	1.67 (1.10 to 2.53)
7	2.42 (1.56 to 3.74)	1.77 (1.11 to 2.82)
8	2.66 (1.60 to 4.42)	1.87 (1.09 to 3.21)
9	2.92 (1.61 to 5.29)	1.98 (1.06 to 3.71)
10	3.21 (1.61 to 6.40)	2.09 (1.01 to 4.32)

[a] Model adjusted for age, race, ethnicity, health insurance, marital status, history of alcohol use and smoking, molecular subtype, AJCC clinical stage, lumpectomy, mastectomy, reconstructive surgery, sentinel and axillary lymph node biopsy, and receipt of chemotherapy
[b] The allostatic load score was modeled using a 3-knot restricted cubic spline OR=Odds Ratio; CI=confidence Interval

Supplementary Table 3: Crude and Adjusted[a] Analyses Between Allostatic Load Biomarkers and Postoperative Complications

Biomarkers	Cutoff Units	Crude OR (95% CI)	Adjusted[a] OR (95% CI)
**Alkaline Phosphatase**	> 126 U/L	0.85 (0.37 to 1.99)	0.71 (0.30 to 1.68)
**Albumin**	< 3.5 g/dL	**2.89 (1.45 to 5.78)**	**2.73 (1.34 to 5.52)**
**Serum Creatinine**	>1.3 mg/dL	**1.56 (1.03 to 2.38)**	1.33 (0.86 to 2.05)
**Heart Rate**	>100	1.27 (0.85 to 1.91)	1.30 (0.85 to 1.99)
**White Blood Cell Count**	>11.19 K/uL	0.96 (0.62 to 1.48)	0.85 (0.54 to 1.33)
**Body Mass Index**	<18.5 or >25 Kg/m^2	1.25 (0.96 to 1.63)	1.10 (0.84 to 1.45)
**Blood Pressure Diastolic**	≥90 mmHg	1.06 (0.78 to 1.43)	1.05 (0.77 to 1.42)
**Blood Pressure Systolic**	≥130 mmHg	1.10 (0.87 to 1.40)	1.00 (0.78 to 1.30)
**Blood Urea Nitrogen**	>25 mg/dL	1.51 (0.92 to 2.47)	1.29 (0.77 to 2.15)
**Glucose**	≥100 mg/dL	1.23 (0.98 to 1.55)	1.10 (0.87 to 1.39)

[a] Adjusted for allostatic load, alkaline phosphatase, albumin, creatinine serum, heart rate, white blood cell count, body mass index (BMI), blood pressure diastolic, blood pressure systolic, blood urea nitrogen, and glucose

OR=Odds Ratio; CI=Confidence Interval

Supplementary Table 4. List of Post-Surgical Complications	
**Category**	**Complication**
Technical	Rebleeding/hematoma
	Disruption of the wound
	Seroma
	Axillary vein injury
	Nerve injury
Infectious	Surgical site infection
	Sepsis
	Pneumonia
	Urinary tract infection
Respiratory	Acute respiratory distress syndrome
Cardiovascular	Acute myocardial infarction
	Cardiopulmonary arrest
	Stroke
	Deep vein thrombosis
Urinary	Acute renal failure
